# Nurses’ experiences with continuous vital sign monitoring on the general surgical ward: a qualitative study based on the Behaviour Change Wheel

**DOI:** 10.1186/s12912-022-00837-x

**Published:** 2022-03-14

**Authors:** J. P. L. Leenen, E. M. Dijkman, A. van Hout, C. J. Kalkman, L. Schoonhoven, G. A. Patijn

**Affiliations:** 1 Department of Surgery, Isala, Dr. van Heesweg 2, 8025 AB Zwolle, The Netherlands; 2 Connected Care Centre, Isala, Dr. van Heesweg 2, 8025 AB Zwolle, The Netherlands; 3 Research Group IT Innovations in Health Care, Windesheim University of Applied Sciences, Campus 2-6, Zwolle, 8017CA The Netherlands; 4 Department of Anaesthesiology, University Medical Centre Utrecht, Utrecht University, Heidelberglaan 100, 3584 CX Utrecht, The Netherlands; 5 Julius Centre for Health Sciences and Primary Care, University Medical Centre Utrecht, Utrecht University, Heidelberglaan 100, 3584 CX Utrecht, The Netherlands; 6 School of Health Sciences, Faculty of Environmental and Life Sciences, University of Southampton, University Rd, Southampton, SO17 1BJ UK

**Keywords:** Telemedicine (MeSH), Monitoring, Physiological (MeSH), Vital signs (MeSH), Continuous vital sign monitoring, Telemonitoring, Wearable devices, Nurses, Implementation, Behaviour Change Wheel

## Abstract

**Background:**

To support early recognition of clinical deterioration on a general ward continuous vital signs monitoring (CMVS) systems using wearable devices are increasingly being investigated. Although nurses play a crucial role in successful implementation, reported nurse adoption and acceptance scores vary significantly. In-depth insight into the perspectives of nurses regarding CMVS is lacking. To this end, we applied a theoretical approach for behaviour change derived from the Behaviour Change Wheel (BCW).

**Aim:**

To provide insight in the capability, opportunity and motivation of nurses working with CMVS, in order to inform future implementation efforts.

**Methods:**

A qualitative study was conducted, including twelve nurses of a surgical ward in a tertiary teaching hospital with previous experience of working with CMVS. Semi-structured interviews were audiotaped, transcribed verbatim, and analysed using thematic analysis. The results were mapped onto the Capability, Opportunity, Motivation – Behaviour (COM-B) model of the BCW.

**Results:**

Five key themes emerged. The theme ‘Learning and coaching on the job’ linked to Capability. Nurses favoured learning about CVSM by dealing with it in daily practice. Receiving bedside guidance and coaching was perceived as important. The theme ‘interpretation of vital sign trends’ also linked to Capability. Nurses mentioned the novelty of monitoring vital sign trends of patients on wards. The theme ‘Management of alarms’ linked to Opportunity. Nurses perceived the (false) alarms generated by the system as excessive resulting in feelings of irritation and uncertainty. The theme ‘Integration and compatibility with clinical workflow’ linked to Opportunity. CVSM was experienced as helpful and easy to use, although integration in mobile devices and the EMR was highly favoured and the management of clinical workflows would need improvement. The theme ‘Added value for nursing care’ linked to Motivation. All nurses recognized the potential added value of CVSM for postoperative care.

**Conclusion:**

Our findings suggest all parts of the COM-B model should be considered when implementing CVSM on general wards. When the themes in Capability and Opportunity are not properly addressed by selecting interventions and policy categories, this may negatively influence the Motivation and may compromise successful implementation.

**Supplementary Information:**

The online version contains supplementary material available at 10.1186/s12912-022-00837-x.

## Background

Serious unexpected adverse events and complications occur regularly on general surgical wards, especially in the group of high-risk postsurgical or elderly frail patients [1–3]. On general wards the current standard of care is intermittent monitoring of vital signs with Early Warning Scores (EWS), in which nurses play an important role in the measurement, recognition of possible deterioration, and follow-up [4]. Common used scores are the New EWS (NEWS) in the UK and the Modified EWS (EWS) in Continental Europe and the USA. However, important limitations of these scores are their intermittent nature and the optimal measurement frequency remains unknown [5–8]. This potentially results in delayed detection of events and thereby inferior patient outcomes [9].

Over the last few years, wearable, wireless measurement devices, such as smart patches on the chest and wrist worn devices for continuous monitoring of vital signs (CMVS) of patients have become available for ambulant patients on general wards [10]. A systematic review about these devices mostly found studies reporting technical validation and feasibility outcomes [11]. Several of these studies reported a broad range of acceptability rates of nurses in working with CMVS devices [12–17]. We also found moderate rates on usability and satisfaction by nurses in our recent feasibility study with the *SensiumVitals®* CMVS system on our general surgical ward [18]. It is important to recognize that implementation of CMVS can only be successful if nurses are able to integrate this technology in routine patient care work flow [19, 20]. Importantly, only when successful implementation in nursing care has been realized, one can reliably investigate the potential effect on patient outcomes and value.

### The Behaviour Change Wheel

**Fig. 1:**
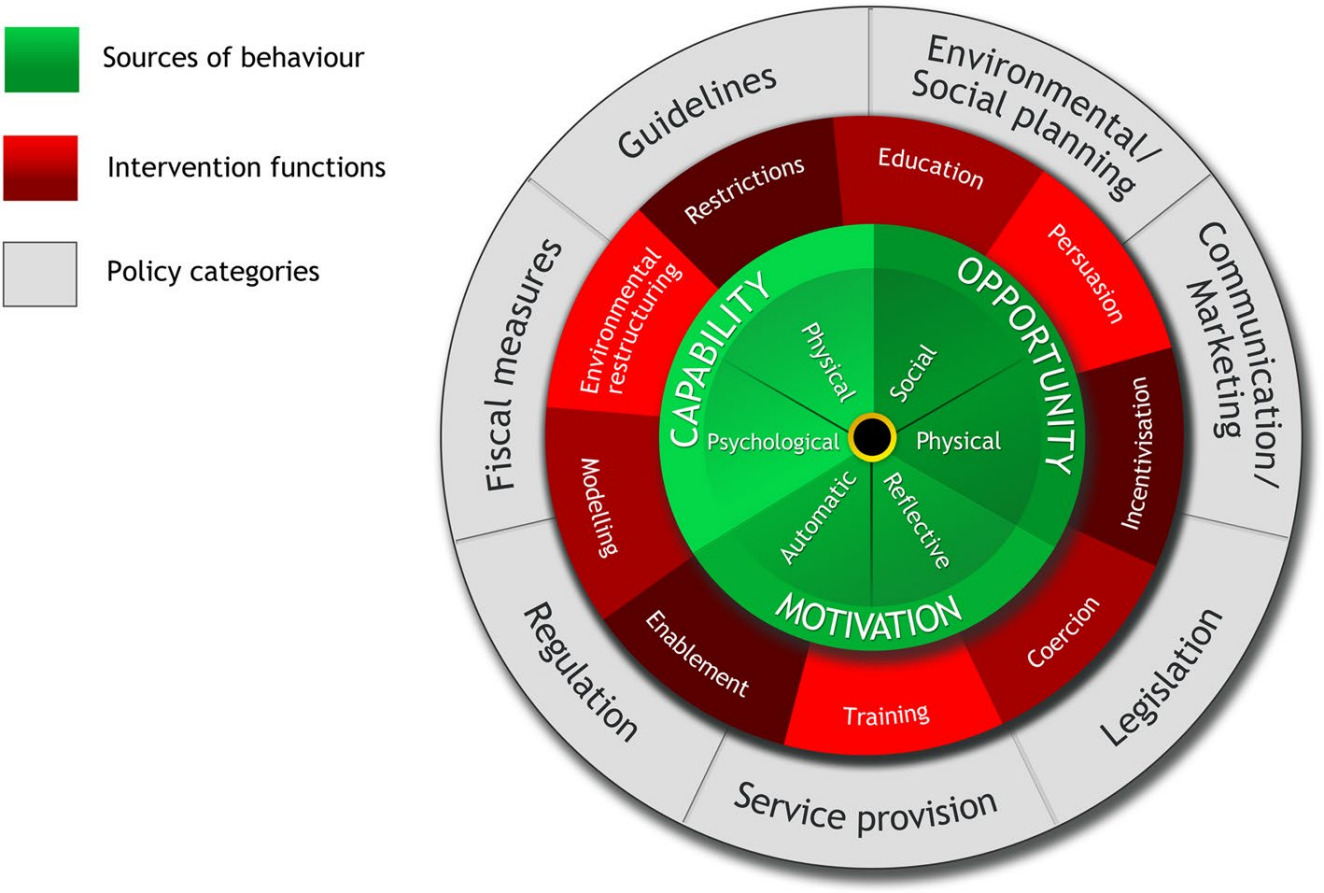
The Behaviour Change Wheel [21]

To guide intervention development and implementation of a CMVS system on the general ward a systematic evidence based approach is needed, such as the Behaviour Change Wheel (BCW) (Fig. 1) [21]. The BCW enables selection of interventions that influence behaviour, which needs to change to enable and support implementation.

**Fig. 2:**
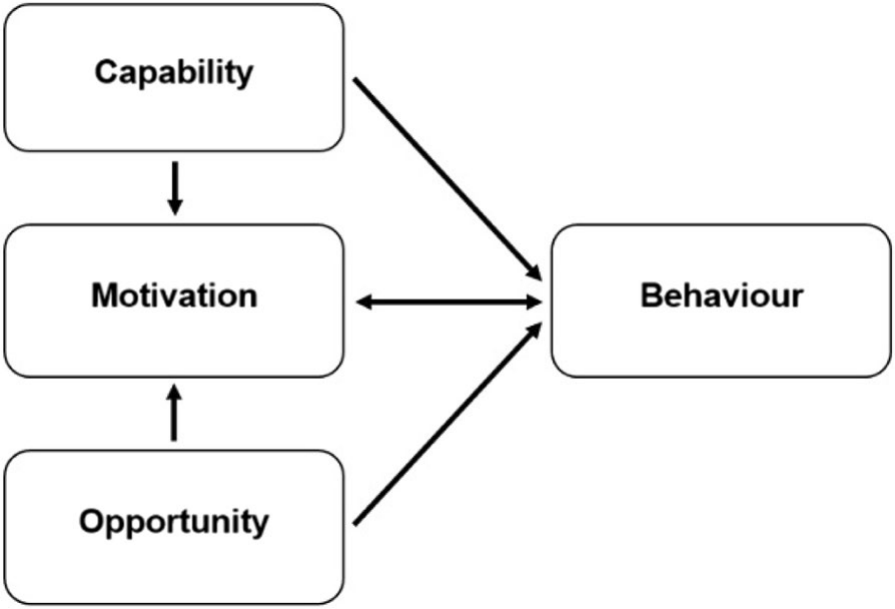
The COM-B model [21]

The core layer of the BCW is the Capability, Opportunity and Motivation model (COM-B) (Fig. 2) [21]. According to the COM-B model, behaviour is part of an interacting system of the social and physical factors. For an individual nurse to engage in a specific behaviour (B) there is a need for ‘capability’ (C) to do it, both psychological and physical. There must also be the social (e.g., support from others) and physical (e.g., the necessary resources) ‘opportunity’ (O) to perform the behaviour. And finally, there must be sufficient strong ‘motivation’ (M) to undertake the desired new behaviour over other competing behaviours. Motivation covers automatic processes involving emotional reactions, desires and impulses, as well as reflective processes involving self-conscious planning and beliefs about what is good and bad [22]. Also, Capability and Opportunity may have an influence on Motivation in the model.

Understanding these factors helps to determine which COM-B components needs to shift for the desired behaviour to occur. After this behavioural diagnosis, the BCW identifies intervention functions and policy categories likely to be effective in bringing about change [22]. So, by defining the COM-B, effective interventions can be selected to address behaviour.

Published studies about CMVS monitoring so far mainly assessed nurses’ experiences with acceptability questionnaires [15, 16, 18]. There is a lack of more in-depth insight in the opinions and experiences of this important stakeholder group for the implementation of CMVS. Therefore, the aim of this study is to provide insight in the capability, opportunity and motivation of nurses providing CMVS, in order to inform and support future implementations using the BCW.

## Methods

### Design

A qualitative study design was applied utilizing semi-structured interviews. This study is reported in concordance with the Consolidated criteria for reporting qualitative research (COREQ) [23].

### Recruitment and participants

All nurses (*n* = 35) who worked with the *SensiumVitals®* CMVS system in a previous feasibility study on a general surgical ward of Isala, a large tertiary teaching hospital in the Netherlands, were eligible to be interviewed [18]. In our previous study, 30 postoperative abdominal patients were continuously monitored over a three month period resulting in 1–4 simultaneously monitored patients of a total of six patients per nursing shift. When passing vital signs thresholds, alarms were sent out to the nurses on a mobile device. These thresholds were based upon the conventional MEWS thresholds [3]. After receiving a vital signs alert, the nurses were asked to measure the patient’s vital parameters manually in accordance with the routine hospital policy; measuring all parameters for a MEWS score. At the end of study, nurses were asked to complete the Usefulness, Satisfaction, and Ease of use (USE) questionnaire.

To explore the nurses’ views and judgments about CMVS, we subsequently interviewed a purposive sampled group of nurses. Maximum variation sampling ensured inclusion of a broad range of perspectives. Recruitment continued until maximum variation was met for age, work experience, the median score on the USE questionnaire or non-response on the questionnaire in the previous study. Sampling based on the USE questionnaire scores was divided in positive (score 4.6–7.0), negative (score 1.0–3.4) or neutral. (3.5–4.5 score) [24]. Eventually twelve nurses were approached and agreed to participate in the interviews with a median duration of 37.5 min (IQR 33.80-IQR 46.36). All respondents were female with a median age of 27.5 (IQR 23–31.5) years old and a median of 5.5 (IQR 2–8.5) of years’ work experience. A broad range of responses on the USE questionnaire of the previous study was represented, namely positive (*n* = 5), neutral (*n* = 3), negative (*n* = 2) and non-response (*n* = 2). The selected participants were approached by email by JL. After explaining the goal of the study and the voluntary participation, informed consent was gained and an interview was scheduled. At the start of the interview, the researchers were not aware of the interviewee’s score on the USE questionnaire to prevent confirmation bias. No new themes emerged after interviewing ten participants.

### Data collection

In preparation for the study, the interviewers (JL; male and ED; female) were trained in qualitative research methods. Both interviewers were part-time employed as nurses at the same ward where the CMVS system was implemented and they knew the nurses before the interviews. Semi-structured, face-to-face interviews were conducted with the nurses at the hospital in a secluded office on the ward between April 2020 and August 2020.

The 25 interview questions were divided over the three elements of the COM-B model (see Additional File 1). The topic guide was developed by three researchers (JL, ED and GP), pilot tested with one ward nurse, and revised during the iterative process of data collection and analysis. The interviewer was guided by the topic scheme, but was allowed to change the sequence of questions within the topics or to add questions for emerging topics. Different probing techniques such as remaining silent, echoing, and asking for elaboration were used to gain further insight into experiences [25].

All interviews were audio-recorded and transcribed verbatim. Keynotes were used to record feelings and thoughts of the researcher [26].

### Data analysis

The interviews were analysed using deductive thematic analysis using the qualitative data analysis software *NVivo 11* (QSR International, London, UK). The raw data was analysed using a six-stage thematic analysis as outlined by Braun and Clarke [27]. The stages include: (1) immersion; (2) generating initial codes; (3) searching for and identifying themes; (4) reviewing themes; (5) defining and naming themes; and (6) writing the report.

Stage 1 to 3 were conducted independently by two researchers (JL and ED). During the first and second stage, JL and ED became familiar with the data by listening to the audio recordings, checking the transcriptions against the audio recording, reading, listening sections again and re-reading the final transcripts. During the third stage, both researchers read the transcripts and codes for categorizing similar statements into first themes.

For the fourth and fifth stages, JL, ED, AvH and CK were responsible for reviewing, defining and naming themes, which were discussed with the other authors. AvH is an expert in qualitative research. Eventually, in the sixth stage the themes were mapped to the COM-B model and discussed with all authors. During the sixth stage, the themes were brought to the nurses for member checking by e-mail, which did not result in any changes to the themes.

## Results

The analytical process resulted in five key themes: learning and coaching on the job, interpretation of vital sign trends, added value for nursing care, management of alarms and integration and compatibility with clinical workflow.

### Learning and coaching on the job

All of the nurses indicated that receiving training and education is conditional to acquire adequate knowledge of the system and to be able to start with CMVS. The preferred educational methods were training sessions, such as an e-learning module, but also information by e-mail. Also, the timing of training and dosage of the amount of information was considered important, preferably shortly before the start of the implementation and repeated regularly during implementation to keep their acquired knowledge up to date. Some nurses who were not trained expressed feelings of insecurity in using the system. However, these feeling were also present in nurses who had gained knowledge by the training. One nurse stated:

‘In the beginning I had to get used to it for a while and I still felt insecure about some aspects of continuous monitoring. But it did help that we just started doing it and having an involved project leader and key users. There was always an opportunity to ask questions and she was also often present in the department, so that you just became really confident in working with it.’ (R15).

Several nurses believed it was important to develop skills in CMVS by handling it in daily practice, the learning on the job. Further, supportive for learning on the job, some nurses mentioned to prefer a printed guideline but, more importantly, coaching by the project leader or from key users and colleagues on the ward. During their shift, key users provided information and instructions to the nurses. One nurse mentioned:

*‘I think that you should also give proper education and training beforehand. But also *providing* extra training for the people who find it difficult in advance. For example, by setting up a personal coaching plan for the nurse. So, you really have to spend time on one-on-one guidance in the first period, so that nurses feel heard. (…) To be able to ask questions about your patient with continuous monitoring to a colleague who knows the system well, that will get you going.’ (R10).*

Several nurses indicated that education before the start of the vital signs monitoring in practice, does not work without applying the new knowledge at the bedside. In particular, practical skills such as pairing the patient to the platform or attaching the patch sensor to the patient are best taught at the bedside. One nurse stated:

*‘To be honest, we had training before the start, but that did not really take root at the time. At the start of the implementation, I really think it would be difficult to work with continuous monitoring. Because you really need the experience in real-life practice, with real patients, if you want to be able to work with this new device properly.’ (R4).*

Also, some nurses indicated that it required some time to gain the practical skills and get used to the new work process. Several nurses mentioned that only a few patients had CMVS instead of all of them during their shifts. As a result, working with two work processes for vital sign monitoring was difficult, confusing and sometimes experienced as extra work. Therefore, they would prefer a higher patient volume of CMVS in the study. One nurse stated:

‘*Yes, continuous monitoring is something that if you want to perform well, I think you really should do it structurally. And I mean, just really work with the system every day with every patient. Not only with some of your patients. Then you will easily learn the system during a few shifts, just in your daily work.’ (R1).*

In summary**,** nurses favoured learning CMVS by actually dealing with such systems in daily practice. An important success factor was that guidance and coaching was available during the initial period of implementation.

### Interpretation of vital signs trends

All of the nurses mentioned their experience with interpreting and judging vital sign trends, but their perspectives varied. On one hand they indicated they were able to assess the trend properly, and on the other hand some nurses experienced difficulty because of the lack of knowledge of what normal trends should look like. Also, the pre-specified vital signs thresholds were guiding in the interpretation, but deviating or irregular trends within the thresholds were challenging to interpret in combination with the clinical status of the patient. Difficulty was also experienced when there were invalid or missing measurements in the trend. One nurse said about this:

*‘I think it is quite hard in the beginning, because you do not know what a vital sign trend should look like. Especially when taking the patient status, activity and missing data in the trend into account. Those factors are important to consider when assessing the trend.’(R6).*

For interpreting the vital sign trends, several nurses thought a clear protocol would be useful. They especially experienced challenges in clinical decision support and follow-up of alarms, because it was unclear what the follow-up actions should be when one vital sign deviated. Also, they found CMVS to be a supplement to current vital signs protocols, mainly because they strongly feel that the full range of vital signs is needed to measure an Early Warning Score. They indicated that measuring more vital signs, provided a more complete insight in the clinical status of the patient. Also they found some specific causes of clinical deterioration are detected by other vital signs, such as blood pressure or body temperature. Therefore, the more vital values are continuously measured, the more complete and informative the scores will be for nurses and physicians. A nurse said about this:

*‘Nowadays we work with the Early Warning Scores. Those are recognizable and guiding in our follow-up actions, like calling a physician when a score is 5. The trends and thresholds did not provide such clear follow-up. Also, because continuous monitoring still does not measure all the vital signs to generate a proper EWS.’ (R2).*

Some nurses considered the collaboration with physicians vitally important for successful interpreting the trends and the follow-up. They thought physicians have more knowledge and experience in trend assessment and should play a major role in the follow-up of deviating trends. They believed the physician has the responsibility to determine medical policy in the event of clinical deterioration. Also, some nurses said it was a shared responsibility of the nurse and physician and that close collaboration is important in vital sign monitoring. For example, one nurse said:

*‘Besides trend assessment by us as nurses, physicians must be involved. They need to know how to act based on deviating trends. Eventually, they are responsible for the medical policy following the trend’ (R3)*

Within their reports on the trend, the nurses placed trends in the perspective of their clinical assessment. One nurse stated:

‘*Yes, I think I should see continuous monitoring as a helpful tool. I don't see it as a substitute for me as a nurse, like: “Oh that one patient has a wireless vital sign monitor and I can blindly rely on those measurements”. But your own clinical assessment of the patient besides vital signs remains most important. For example, if you observe values measured by the device, it is important that you always use your own observations as a nurse and decide whether it fits the patient’s condition.’ (R7).*

Also, most of the interviewed nurses mentioned they had no experience with a clinically deteriorating patient with a continuous vital sign monitor during this study period. They thought this would be helpful to learn to interpret the vital sign trends. A nurse said about this:

*'I think it is helpful if you cared for a patient that had an acute clinical deterioration. Then you possibly have a clear picture of such a deviating vital sign trend in combination with the clinical status of the patient.'*

This statement relates to the previously mentioned theme learning and coaching, on which several nurses mentioned learning in practice with real patients was important for successful use of the CMVS systems. Further, nurses believed that CMVS could support their clinical reflection and judgment during their work, although several believed that their overall clinical assessment of the patient was important for the evaluation of trend monitoring, and that technology alone cannot be relied upon for clinical decision making.

### Added value for nursing care

All nurses recognized the potential added value of CMVS for postoperative nursing care based upon their experience in practice. They considered vital signs as an important element of clinical evaluation on the ward and believed this technology may contribute to earlier detection of clinical deterioration by better insight into the vital sign trends and thus increase the safety of care. One nurse stated about this:

*‘I think it can offer a lot for us and patients, especially if you are able to detect the complications earlier. By the insight in trends you may detect clinical deterioration earlier between the routine measurements.*

*In addition, in the end that you also get less intensive care unit (ICU) admissions or patients who spend less time on the ICU.’ (R6)*

Also, several nurses thought that CMVS may only prove to be beneficial for patients with a high risk of clinical deterioration, for whom the benefits of rapid recognition of acute deterioration are most obvious. They considered there should be a clear rationale to measure vital signs at a high frequency. Otherwise, they considered current manual measurement intervals to be sufficient. A nurse said:

*‘I would not see much added value for low-complexity care. These patients already have a low risk of complications and so clinical deterioration of vital signs. For example, consider an appendectomy.’ (R1)*

In relation to this statement, the same nurse also mentioned that the costs of implementation of CMVS systems should be in proportion to the benefits for patient care. High costs for the implementation and for the purchase of software or hardware should be justified by a reduction in the cost of care through a decrease of complication rate, length of stay, ICU admission or readmissions. A nurse said:

*‘If the wearable sensor is very expensive, it is worth considering whether the investment is worth it for the particular patient group. I do not think it is effective to apply on those low-complex care patients.’ (R1)*

Besides, having ability of continuous insight in the patient vital signs, the nurses found the possibility of remote monitoring of the patient especially useful during night shifts because of the higher patient-to-nurse ratio. Also, one nurse mentioned there is a desire not to unnecessarily wake the patient. A nurse said:

*‘During the night shift you have a direct insight and an overview whether each patient is still breathing or showing abnormalities in vital signs. This is really helpful when you nearly have a half ward of patients to take care of.’ (R11).*

Overall, nurses believed in the potential added value of CMVS to increase the safety of care by earlier detection of clinical deterioration by better insight into the vital sign trends.

## Management of alarms

Most nurses mentioned their experience with the alarms generated by the CMVS system. All of them experienced that the system generated too many and too many false alarms. This was possibly caused by the system’s set time frame of only fifteen minutes for sending out alarms. Besides, the false alarms were mainly caused by the system’s strict artefact rejection algorithms for respiratory rate and motion artefacts. These alarms were experienced as disruptive and caused feelings of uncertainty and lead to irritation. One nurse said:

*‘I found the number of alarms that you got on your telephone the most inconvenient for me. There were really too many. This was often already with a deviation or technical problem for a short time. For instance, when you support in mobilization, you don't have time to check the notification on your phone every time. You can't leave the patient at all at that moment so an alarm does not add up to better care.(…) Sometimes I was happy when the alarms didn't ring for a while.’ (R1).*

This quote reveals feelings of possible agitation about the alarms, potentially related to the extra workload caused by the need to respond to the alarms. Also, feelings of uncertainty raised by alarms were caused by having doubts about their own clinical experience by receiving multiple and frequent alarms. They also mentioned that many alarms and the relatively high rate of false alarms also indirectly may have bothered the patients because of the necessary extra checks conducted at the bedside. Nurses suggested user-adjustable alarm settings to decrease false alarm rate and prevent alarm fatigue. One nurse said about this:

*‘Often as a nurse you could not do anything with the alarm because the heart rate had already dropped again or the connection had already been restored. Then you start doubting whether you are doing your work right or not missing any abnormalities in the patient condition. (…) Also, adjusting values to the specific patient could be helpful in reducing alarms.)’ (R5).*

In summary, the quantity and frequency of (false) alarms generated by the CMVS system were experienced as excessive. This resulted in feelings of agitation and uncertainty, when they were unable to directly respond to the alarms. In addition, they mentioned that the availability of continuous monitoring on the ward should not be a reason to consider this type of vital sign monitoring to be similar to an ICU setting.

### Integration and compatibility with clinical workflow

Nurses found CMVS easy to use overall. However, working with CMVS and the integration in nursing practice was influenced by a number of factors.

Several nurses preferred a CMVS system technically integrated into their existing mobile devices without restrictions in the range of the wireless connection. Also, they strongly favoured integration of vital signs trends into the Electronic Medical Record (EMR) allowing more effective documentation, evaluation and productivity. A nurse said:

*‘It does work better for me if we can assess the trends in the current used systems such as the EMR, but also receiving alarms on the calling system instead of using a separate phone. This makes everyday use much easier’ (R7).*

Further, two nurses mentioned that availability of CMVS should not be a reason to discharge patients earlier from the ICU to the ward. They expressed certain fears that this might result in a higher workload and unsafe nursing care. A frequently mentioned reason was the inability to immediately respond to alarms as reported in the previous theme. This also highlights that the focus on and importance of vital signs monitoring is perceived differently by general ward nurses and ICU nurses. One nurse said:

*‘If an alarm rings from one patient and at the moment you are bathing a patient and you also have to care for four other patients, then responding to the alarm can be challenging. I think that's different on an ICU.’(R9)*

Other mentioned reasons relating to clinical workflows were the current high workload at their ward because of the lower nurse-patient ratio. Also, they believed not to have the technical nursing skills and knowledge of vital signs monitoring that ICU patients would need. One nurse said about this:

*‘Continuous monitoring should not be a reason for patients to be discharged from the ICU to our ward earlier. We care for many more patients per nurse and in case of acute deterioration we do not have the same resources. It then becomes impossible to provide good quality care. Maybe even dangerous for patients.’ (R9)’*

Several nurses also expressed the hope that in the future CMVS devices will be able reduce the workload of current routine manual measuring and registering vital signs, allowing them to be more productive and have more dedicated time for patient care. One nurse said:

*‘I hope in the future wearable sensor will measure the full spectrum of vital signs so I don’t have to collect them manually several times a day. This will save time which I can still devote to many other tasks during a busy shift.’ (R5).*

Overall, CMVS was experienced helpful and easy to use, although several improvements were mentioned such as integration in mobile devices and EMR and the need to securely manage clinical workflows and protocols when transferring high-risk patients from the ICU.

### Themes in relation to the COM-B

The five generated themes were mapped onto the COM-B model (Table 1). Two themes related to Capability and two themes were related to Opportunity. All themes had a relation to Motivation. One theme was linked to Motivation.

**Table 1:** Themes mapped onto the COM-B model

Theme	COM-B component
Learning and coaching on the job	Capability, Motivation
Interpretation of vital signs trends	Capability, Motivation
Management of alarms	Opportunity, Motivation
Integration and compatibility with clinical workflow	Opportunity, Motivation
Added value for nursing care	Motivation

## Discussion

To our knowledge, this is the first study providing an overview of nurses’ perceptions of behavioural factors that influence implementation of a CMVS system on general surgical wards. Application of the COM-B model provides a theoretical framework for understanding nurses’ views and behaviour in CMVS systems on the ward and may guide in selecting the relevant interventions and policy categories of the BCW. Using semi-structured interviews five relevant themes were identified a related to nurses’ capability, opportunity, and motivation, which were mapped onto the COM-B model. As expected, themes within Capability and Opportunity were also potentially influencing Motivation.

Considering Capability, it was evident that nurses must be adequately trained before starting to work with the CMVS system. However, for successful implementation, bedside learning and coaching to enhance their knowledge and skills in clinical practice, seem to be important for nurses. The desire of developing skills and training with support and coaching during implementation of CMVS was also reported in other studies [12, 15]. Although it seems that this type of learning may be most appropriate, it is also advised to offer other types of learning methods to match the various learning style preferences as well as take into account the variation in attitudes towards innovation [28, 29]. Related to this, nurses perceived that a certain minimum volume of patients with CMVS on the ward is needed to build routine. Nurses consider this essential, especially in the initial phase of the implementation which is in line with previous findings that eHealth acceptance requires sufficient time and exposure by a high patient volume [30].

The capability of nurses to interpret vital signs’ trends was also important. Nurses mentioned assessing trends instead of the standard absolute EWS values was challenging. This is in line with statements of physicians about nurses not having adequate training to interpret continuous data in an earlier study [31]. Besides training, developing adequate trend interpretation skills is expected to take a high patient volume and specific exposure to clinically deteriorating patients with CMVS, which was limited in this study.

Moreover, nurses’ overall clinical assessment, obtained by direct patient contact and based on their professional experience, should be incorporated into the evaluation of vital sign trends. Obviously, nurses’ observations on the patient status and possible clinical deterioration is much more than just monitoring vital signs. Current sensors and vital sign trends still do not include factors such as the nurse worry factor and the critical EWS component ‘level of consciousness’ [32–34]. In line with other studies, the value of the nurse’s clinical observations in detection of deterioration was also with respect to reservations about a potential decrease in the bedside nurse-patient contacts by using CMVS which may limit the value of their clinical judgement [15, 35, 36].

Also, nurses strongly valued the role of the physician in trend assessment because of their expertise with vital sign trends interpretation as part of their clinical judgement. Besides, they thought physicians should play a role in the follow-up of the trends. This may also be a relevant factor for implementation of such systems, which was mentioned in a previous study, in the context that CMVS may support interdisciplinary communication between nurses and doctors [12].

Considering Opportunity, nurses generally believed that CMVS may fit well into their clinical workflow, which was also recognized in other studies [31, 37]. Although, we found that smooth integration in IT systems and clinical workflows as well as selective alarm management are important factors to support successful CMVS implementation. Specifically, this includes the need for CMVS data integration into the EMR and in mobile devices and an adequate connectivity and range of the sensor, which was also mentioned in previous studies [11, 36]. Also, integration in clinical workflows should be optimized. Especially, clear criteria to prevent premature transfers of patients from ICU to the general ward with CMVS are needed, which was also was mentioned as a potential worry in another study [32].

Importantly, the multitude of (false) alarms in our study was perceived as excessive, which may cause alarm fatigue and may be a major barrier for successful implementation. In several other studies, nurses also reported frequent (false) alarms to be the biggest disruptive factor for their work processes [31, 38], although in one study nurses found alarms were generally appropriate [16]. Currently, alarm strategies used by CMVS systems are mostly based on conventional high or medium care unit protocols, using pre-set thresholds values. However, this does not consider other factors such as the delta of trends over time, the mobilization of the ambulant patient on general wards, and circadian rhythm of the patient. Therefore, for general wards more sophisticated alarm strategies would be desirable, but these are still under development [39]. Alternatively, strategies relying on routine trend assessments only (e.g. several times per day) rather than using pre-set alarms may be a solution to deal with excessive alarms and support implementation and compliance on general wards.

Considering Motivation, nurses seem to be clearly motivated to use this innovation because they believe in the potential for improving the quality and safety of patient care. The potential benefit for patients was also recognized by nurses in several other studies with a CMVS systems, specifically for earlier detection of clinical deterioration in certain high risk patient groups and providing remote insight in the patients vital signs during night shifts [14, 31, 32, 36]. Unfortunately, contrary to common belief among nurses strong evidence for clinical benefit and cost-effectiveness is still lacking due to the various study designs, low study quality and various outcome measures used in available published reports [11, 40]. However, providing nursing care according to the principles of Evidence-Based Practice is more than just the following the evidence, but also consists the preferences of the patient and clinical expertise of the nurses [41].

Taken all together, based on the five themes identified and subsequent mapping onto the COM-B model, several intervention functions of the BCW may be applied to allow successful implementation (Fig. 1) [22]. Bedside training and education could enhance the Capability of nurses about CMVS. Enablement and environmental structuring may address the themes mapped onto Opportunity as described above. Lastly, modelling may strengthen the Motivation of nurses. Supporting to the intervention functions, the possible policy categories of the BCW could be guidelines, environmental planning and legislation.

## Limitations

The findings in this study need to be interpreted in light of several limitations. First, our study was performed on a Dutch general surgical ward which may affect transferability to other countries and specialisms. Also, the experience of nurses was with one particular CMVS platform (*SensiumVitals®*), while many other systems are available [11, 42]. However, we emphasized beforehand to respondents that we wished them to give us their opinion on the concept rather than the particular system we used. Furthermore, we only included female nurses in our study so results may not be transferable for male nurses. However, a previous study did not show a significant effect on technology acceptance between genders [43]. Moreover, respondents’ experience with CMVS was based on a relatively short period of working with the new system and a limited number of patients per nursing shift, whereas sufficient exposure is a known condition for successful implementation of innovations. Also, the extensive interview guide gave a broad overview of the nurses’ perceptions but limited in-depth insights. Moreover, framing of the themes to the COM-B and BCW model may have limited the openness of the interviews as other frameworks such as the Technology Acceptance model are not considered [44, 45]. However, the COM-B model does take the challenging context factors on the ward into account. Finally, JL and ED were part-time employed as nurses at the same ward where the CMVS system was implemented. Although it was explicitly stated that answers had to be given honestly, this may have influenced the social desirability of the answers. On the other hand, the interviewers had a broad experience in clinical nursing, qualitative research methods as well as the technical aspects of CMVS. This supported the understanding of the context and quality of the study design. Another strength of this study was that the application of analyst triangulation by coding and forming and framing themes was done independently by several authors (JL and ED).

## Conclusion

CMVS using wearable wireless devices may support the timely detection of clinical deterioration. Successful implementation of such novel technology is important but challenging. This study provides an overview of the nurse experiences regarding the implementation of CMVS on a general surgical ward. Our findings suggest all parts of the COM-B should be considered when implementing CVSM on general wards, with particular attention to the complexity of interaction of the elements of the model. When the themes in Capability and Opportunity are not properly addressed in the selection of interventions and policy categories, this may negatively influence the Motivation and may compromise successful implementation.

Collectively, our findings related to the COM-B model may guide implementation strategies of CMVS systems on general wards when using the intervention functions and policy categories of the BCW. Further studies should focus on evaluation of implementation strategies of such systems in daily practice.

## Supplementary Information

**Additional file 1. **

## Data Availability

All data generated or analysed during the current study are available from the corresponding author on reasonable request.

## Abbreviations

BCW: Behaviour Change Wheel
COM-B: Capability Opportunity Motivation – Behaviour
EMR: Electronic Medical Record
IT: Information Technology
USE: Usefulness, Satisfaction, and Ease of use questionnaire
