# Supplementary material for: Nurses’ experiences with continuous vital sign monitoring on the general surgical ward: a qualitative study based on the Behaviour Change Wheel
Source: BMC Nurs. 2022 Mar 14;21:60. doi: 10.1186/s12912-022-00837-x (PMC8919550; doi:10.1186/s12912-022-00837-x)
Supplement: Supplementary file 1 — Additional file 1. [file 12912_2022_837_MOESM1_ESM.pdf]

Additional File 1: Interview guide (translated from Dutch)

**CAPABILITY**	
Psychological Capability	
Knowledge	Did you have sufficient understanding of the possible benefits of continuous monitoring?<br>Were you familiar with any guidelines or policies during the study? Can you describe what the guidelines or policies say?
Behavioural Regulation	What do you think is needed to ensure that you consistently provide continuous monitoring to patients?
Memory, Attention, and Decision Process	Are there situations when you think it would be difficult to provide continuous monitoring to patients? (prompt – can you tell me what it is about these situations that make it difficult)
Physical Capability	
Skills	Did you feel you have the skills to provide effective continuous monitoring to patients?<br>(prompt –are there any other skills that you need?)<br>What skills do you think are needed to provide effective continuous monitoring to patients?
**OPPORTUNITY**	
Social Opportunity	
Social influences	Did you ever discuss continuous monitoring or policies with other nurses at your ward?<br>Did other nurses at your ward influence your decision to provide continuous monitoring to patients? How would they influence your practice? To what extent?<br>Do your colleagues value providing effective continuous monitoring to patients?
Physical Opportunity	
Environmental Context and Resources	What factors outside of your professional/practice environment influenced your ability to provide more effective continuous monitoring?<br>Were there competing tasks or time constraints that would influence your ability to provide more effective continuous monitoring?

**MOTIVATION**	
Automatic Motivation	
Reinforcement	Were there any incentives for you to provide continuous monitoring? What are they? When you provide continuous monitoring to patients do you feel like you are making a difference? Why or why not?
Emotion	Does discussing continuous monitoring ever evoke an emotional response in you? (prompt – would you feel worried or concerned about providing continuous monitoring?)<br>Thinking about yourself and how you normally feel as a professional that works with patients, to what extent do you feel inspired to provide continuous monitoring?
Reflective Motivation	
Social/Professional Role And Identity	What responsibilities did you have as a nurse to provide continuous monitoring at the ward?<br>How was continuous monitoring consistent or inconsistent with your profession?<br>How compatible was the provision of continuous monitoring with your profession?
Beliefs About Capabilities	How confident did you feel in your ability to work with continuous monitoring at your ward?<br>How easy or difficult was it to provide continuous monitoring at your ward to patients?<br>What would made it easy or difficult for you?
Beliefs about Consequences	Do you find continuous monitoring at your wards useful?
Optimism	How optimistic are you about the future of continuous monitoring on the general surgical ward?
Intentions	On a scale of 1 to 10 and 10 being very important, how important do you think it is for you to provide continuous monitoring to patients at your hospital? Why?
Goals	Would the goal of improving and implementation continuous monitoring at your wards be compatible with your usual practice? Why?
